# Effects of *Platycodon grandiflorum* on Gut Microbiome and Immune System of Immunosuppressed Mouse

**DOI:** 10.3390/metabo11120817

**Published:** 2021-11-29

**Authors:** So-Yun Jhang, Sung-Hyen Lee, Eun-Byeol Lee, Ji-Hye Choi, Sohyun Bang, Misun Jeong, Hwan-Hee Jang, Hyun-Ju Kim, Hae-Jeung Lee, Hyun-Cheol Jeong, Sung-Jin Lee

**Affiliations:** 1 Interdisciplinary Program in Bioinformatics, Seoul National University, Kwan-ak Gu, Seoul 08826, Korea; soyun4595@egnome.co.kr; 2 National Institute of Agricultural Sciences, Rural Development Administration, Iseo-myeon, Wanju-Gun 55365, Korea; dmsqufdl1029@naver.com (E.-B.L.); jyyye@naver.com (J.-H.C.); rapture19@hanmail.net (H.-H.J.); 3 Institute of Bioinformatics, University of Georgia, Athens, GA 30602, USA; sohyunbk@gmail.com; 4 eGnome, Seoul 05836, Korea; soonielove33@gmail.com; 5 World Institute of Kimchi, Nam-Gu, Gwangju 61755, Korea; hjkim@wikim.re.kr; 6 Department of Food and Nutrition, Gachon University, Seongnam 13120, Korea; skysea1010@gmail.com; 7 Food R&D Center, Hyundai Bioland Co., Ltd., Manhae-ro, Danwon, Ansan 15407, Korea; hyuncheolj@hyundaibioland.co.kr (H.-C.J.); sungjinreal@hyundaibioland.co.kr (S.-J.L.)

**Keywords:** *Platycodon grandiflorum*, gut microbiome, metagenomics, *Akkermansia*, microbiome, immune system

## Abstract

*Platycodon grandiflorum* (PG) is a perennial plant that has been used as a traditional remedy to control immune-related diseases. PG was steamed and dried to improve its taste (PGS). The aim of the study was to investigate the effects of PG and PGS (PG-diets) on the gut microbiome and immune system. We treated PG-diets to immunosuppressed mice via cyclophosphamide (CPA) injection. After two weeks of the supplement, we evaluated specific genera related to body weight and serum immunoglobulin levels and analyzed 16S rRNA sequencing and metagenomics statistical analysis. PG-diets groups showed an increased abundance of microorganisms in immunodeficient mice compared to the control group (NC). Moreover, *Akkermansia* significantly decreased in response to the CPA in the NC group at the genus level, whereas its abundance increased in the PG-diets groups. We also found that the modulation of the gut microbiome by PG-diets was correlated with body weight, IgA, and IgM levels. The results demonstrate that PG-diets may improve the health benefits of immunosuppressed mice by altering the gut microbiome, though not much difference was found between PG and PGS treatments. Finally, this is the first study showing the effects of PGS-diets on the gut microbiome and immune system as a potential nourishing immunity supplement.

## 1. Introduction

The gut microbial community is associated with host digestion, nutrition, and regulation of the host immune system [1]. In addition, the microbiome influences the development and function of the immune cells at both mucosal and non-mucosal sites. This affects the immune system through the whole body as well as the gut immune system [2]. Given the association between the gut microbiome and the immune system, the use of prebiotics is suggested as one of the solutions to improve the immune system. Prebiotics are mostly fibers that are non-digestible food ingredients that can beneficially affect the host’s health by selectively stimulating the activity of some microorganisms [3]. Indeed, prebiotics stimulate beneficial bacteria including lactic acid bacteria and bifidobacteria, which may involve modifying gut pH, increasing the expression of anti-inflammatory cytokines, stimulating immunomodulatory cells, and thus enhance the immune system [4].

One of the food ingredients with prebiotic-like effects on the gut microbiome is saponins [5]. Saponins in ginseng (Panax ginseng Meyer) are digested by the gut microbiome, and the substrate produced by microbiome affects the immune system [6]. For instance, ginsenoside, a saponin in ginseng, is transformed to compound K by the gut microbiome. The compound K is absorbed into the blood and exhibits potent pharmacological effects such as antitumor, anti-inflammatory, antidiabetic, antiallergic, and neuroprotective effects [7]. The amount of saponins absorption is higher through the metabolism of the gut microbiome than by direct absorption [8]. Additionally, *Gynostemma pentaphyllum* with abundant saponins boosted the beneficial microbes [9]. Thus, food with enriched saponins is expected to influence the gut microbiome related to the immune system [10].

*Platycodon grandiflorum* (PG), which is known as a herbal medicine in Asia, contains abundant saponins [11]. Specifically, the root of PG has been used to treat various diseases, including bronchitis, asthma, pulmonary tuberculosis, diabetes, and inflammatory diseases [12]. The immune-enhancing effect of PG is mainly exerted by PG root-derived saponins [13]. In previous studies, PG root-derived saponins were found to have anti-inflammatory and anti-oxidative activity [14]. Furthermore, saponins from PG showed inhibitory effects against infection of Hepatitis C Virus [15]. In our previous in vivo experiment in mice, PG improved the immune function by increasing body weight and the serum level of immunoglobulins (IgA and IgM) [16]. IgA and IgM are key factors in the immune system that play a role in neutralizing toxins, bacteria, or viruses [17]. Furthermore, recent studies have reported that PG increased the level of immunoglobulins in cyclophosphamide (CPA)-immunosuppressed rats, which suggested a positive role in enhancing the immune response [18,19]. Therefore, the beneficial effects of PG might be linked with an altered gut microbiome, considering that saponins are associated with the gut microbiome.

We developed an aged red PG (PGS) with 2.6 times higher concentration of platycodin D, which is known as a functional compound in the PG, by steaming and drying the PG. PGS showed improved immune-stimulating effects on the immune-suppressed mice in the previous study [20]. In many studies, functional foods affect host health by improving the microbiome system. However, there is less research or information about PG or PGS (PG-diets) on microbiome changes in the immune-suppressed mice. Thus, this study was conducted to examine its effects on the gut microbiome and immune system in the immune-suppressed mice that are induced with CPA [21]. To validate the effect of PG-diets on the immune system and its association with the gut microbiome, we supplemented PG or PGS to immune-deficient mice. Furthermore, to compare the effect of PG-diets with that of commercial immune-improvement additive, one group was fed by β-glucan [22].

The objective of this study is to reveal the impact of PG-diets with respect to altering microbiome and the immune system. We investigated the changes in the gut microbiome compositions in mice exposed to PG-diets and examined the specific genera related to immune-related biomarkers.

## 2. Results

### 2.1. Effects of Platycodon grandiflorum on Host Immune System and Gut Microbiome Diversity

**Figure 1:**
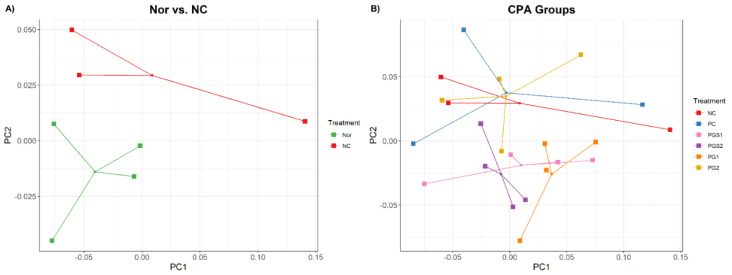
A principal coordinate analysis (PCoA) plot showing dissimilarities of microbiome composition among different diet groups. (**A**) Comparison between Nor and NC using PCoA from the distance of weighted UniFrac. Each dot represents one sample. Green dots are the normal control group (Nor), and red dots are the negative control group (NC) that are immunosuppressed with cyclophosphamide (CPA). (**B**) Comparison of immunosuppressed with CPA groups named NC, positive control (PC), aged red PG (PGS1, PGS2), and PG (PG1, PG2) using PCoA plot.

The principal coordinate analysis (PCoA) with weighted Unifrac distance was performed in two comparisons: (1) between normal control (Nor) group and CPA-treated negative control (NC) group, and (2) between the NC and the groups supplemented by β-glucan (PC), PGS, and PG. In the first comparison, the Nor and NC groups were segregated, but they had high variance within groups (Figure 1A). When we compared NC to other groups that are immunosuppressed with CPA, the positive control with β-glucan (PC) and PG2 groups had no segregation with the NC group (Figure 1B). On the other hand, PGS1, PGS2, and PG1 groups were distinguished from the NC group, where the PGS2 group was clustered at a shorter distance compared with those in the other groups.

Along with PCoA, a nonparametric Permutation Multivariate Analysis of Variance (PERMANOVA) was performed with 999 permutations and used to determine the significant differences between the seven groups. Pairwise combinations with PG1 have shown significantly lower *p*-values than that of the other pairwise combinations, indicating that the abundance and variety of microorganisms in immunodeficient mice exposed to PG1 differed from that of the NC and PC groups. As a result, PG1 can alter the composition similar to the healthy Nor group.

In terms of alpha-diversity, microbial diversity within a local community was evaluated based on species richness and diversity using the observed operational taxonomic units (OTUs) and Shannon index. There was no clear significant difference in both richness and diversity among the seven groups, but richness tends to increase in PG-diets groups compared to the NC group (Figure S1). Overall, the microbial diversity showed a similar distribution across combinations.

### 2.2. Change in Microorganism Abundance by Supplement of Platycodon grandiflorum

**Figure 2:**
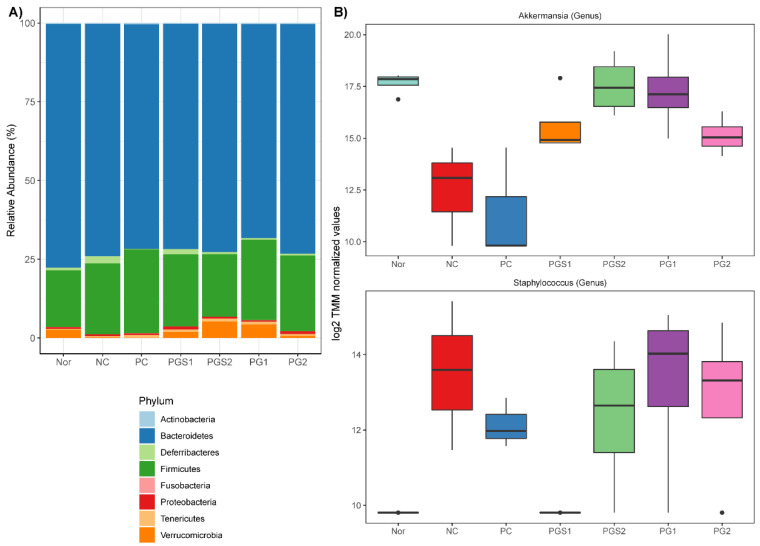
Relative Abundance of microorganisms at phylum and genus levels. (**A**) Phylum level composition. Bar plots represent the percentage (%) of average abundance for each group. (**B**) Differentially abundant values among genus level between control and immunosuppressed groups. Two genera (*Akkermansia* and *Staphylococcus*) were differentially abundant with a significant FDR < 0.05. A dot outside the whisker boundaries is considered an outlier.

The predominant microbes at the phylum level were Bacteroidetes and Firmicutes. They accounted for more than 70% and 20% of microbes (Figure 2A), respectively. The Bacteroidetes, when observed in the NC group, showed a decrease in abundance but an increase in PG-diets. Alongside this, the *Firmicutes* showed a substantial increase in abundance of PG-diets groups. Since only limited information was available regarding the differences at the phylum level, we compared the abundance of the NC group to those of PC, PGS, and PG treated groups to detect supplement of PGS associated microorganisms at the genus level. By using the weighted trimmed mean of the log expression ratios (trimmed mean of M-values (TMM)), the NC group associated microorganisms were *Akkermansia* and *Staphylococcus* (FDR < 0.05) (Figure 2B). The abundance of *Akkermansia* decreased in the NC group compared to the Nor group. When immune-suppressed mice had a supplement with PG-diets, the abundance of *Akkermansia* increased. There were significant differences within the NC group and PGS2 and PG1 groups (*p*-value < 0.05). On the other hand, the abundance of *Staphylococcus* increased in the NC group compared to the Nor group. Dissimilar to *Akkermansia*, there was no distinct difference within the groups, but a decrease in *Staphylococcus* abundance was observed in PC and PG-diets groups. We also carried out the Analysis of Composition of Microbiomes (ANCOM) implemented through the QIIME2 ANCOM plugin to investigate differentially abundant microorganisms at phylum and order levels and compared it with the results of edgeR. Nine phyla were identified, but *Verrucomicrobia* were the only phylum to show a significant change in response to treatments. Moreover, this observation was consistent for the taxonomic classification at the order level, with *Verrucomicrobiales* being significantly different among treatments (Figure S2).

### 2.3. General Characteristics of Phenotypes in Seven Groups

**Figure 3:**
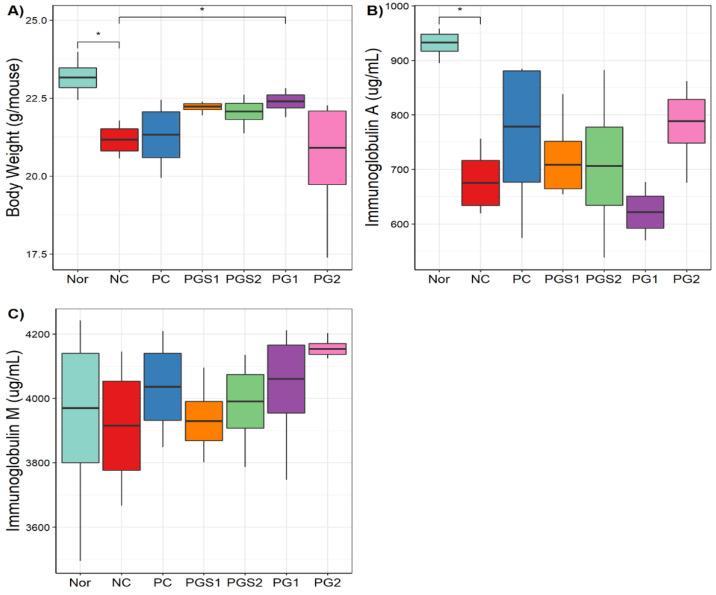
Body weight and serum levels of immunoglobulins (IgA, IgM) of mice. (**A**) A boxplot of body weight in seven groups, (**B**) Boxplot based on immunoglobulin A, (**C**) Boxplot based on immunoglobulin M. The *x*-axis represents the seven groups and the *y*-axis presents measurements of body weight, IgA and IgM (*: significance with *p*-value < 0.05).

Body weights and serum immunoglobulin (IgA and IgM) levels for seven groups were measured in the present study. The Nor group had the highest body weight and IgA (Figure 3A,B). For body weight, the NC group showed a significant decrease compared to the Nor group (*p*-value = 0.0103). The PG1 group showed a significant increase compared to the NC group (*p*-value = 0.049). Body weight increased in the PGS1 and PGS1 groups compared to the NC group (*p* > 0.05). A similar observation was found in IgA, where the NC group showed a significantly lower IgA value than the Nor group (*p*-value = 0.0156). The IgA levels of PGS1, PGS2, and PG2 were higher than that of the NC group (*p* > 0.05). Likewise, PG-diets groups showed higher serum IgM levels than the NC group (*p* > 0.05; Figure 3C). Thus, PG-diets significantly affected body weight in the immunosuppressed mice and tended to increase levels of IgA and IgM, but not much of clear significant differences were found between the NC and PG-diets or within PG-diets groups.

### 2.4. Correlation between the Immune-Related Biomarkers and Relative Abundance of Microorganisms

**Figure 4:**
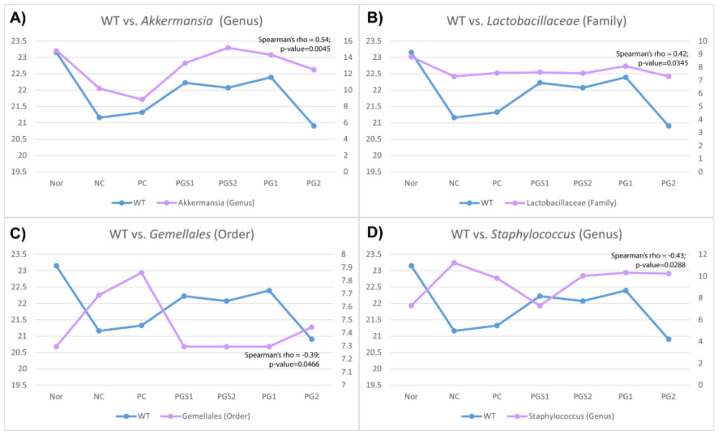
Correlation between immune-related biomarkers and relative abundance of microorganisms. (**A**) A correlation between body weight and genus *Akkermansia*, (**B**) Correlation between body weight and family *Lactobacillaceae*, (**C**) Correlation between body weight and order *Gemellales*, (**D**) Correlation between body weight and genus *Staphylococcus*. *Akkermansia* genus and *Lactobacillaceae* family were positively correlated, and *Gemellales* order and *Staphylococcus* genus were negatively correlated with body weight. The *x*-axis represents the seven groups, the left *y*-axis is body weight value (blue), and the right *y*-axis is the relative abundance value (pink). The Spearman’s rho and the *p*-value for the correlations were shown in the right top corner.

*P. grandiflorum* has been shown to affect body weight, one of the immune-related biomarkers, and the microbiome composition such as *Akkermansia* genus, *Lactobacillaceae* family, *Gemellales* order, and *Staphylococcus* genus (Figure 4). Spearman’s rank correlation coefficient was significant for four microorganisms (Spearman’s rho; *p*-value < 0.05). Among the four genera associated with body weight, *Akkermansia* showed the highest positive correlation, and *Staphylococcus* showed a highly negative correlation with body weight, given 0.54 and −0.43 of Spearman’s rho, respectively (Figure 4). Moreover, six microorganisms were associated with IgA level, *Clostridium* genus, *Adlercreutzia* genus, and *Prevotella* genus found to be positively correlated, and *Mogibacteriaceae* family, *Ruminococcus* genus, and *Aerococcaceae* family were negatively correlated (Figure S3A). In IgM level, *Bacillaceae* genus and *Rikenellaceae* family were positively correlated, and *Mucispirillum* genus, *Prevotellaceae* family, and *Corynebacterium* genus were negatively correlated (Figure S3B).

### 2.5. Functional Pathways of the Microbiome

**Figure 5:**
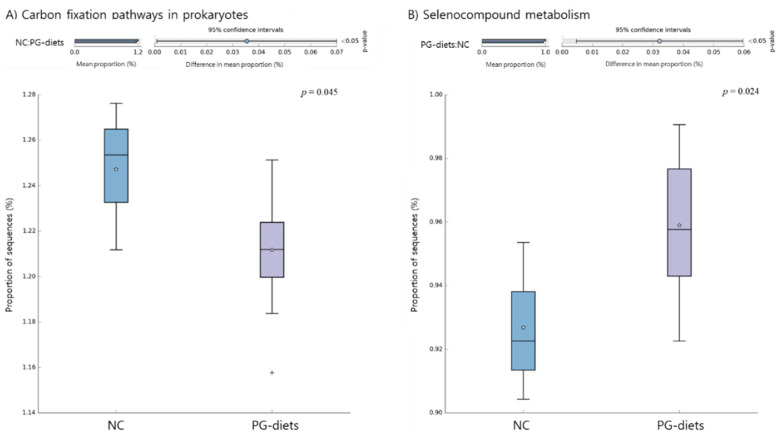
Predicted microbial functions showing significant differences between NC and PG-diets groups. Functions were predicted by PICRUSt2 against the KEGG pathway database, and ANOVA statistical test (*p*-value < 0.05) was performed using the STAMP software program. (**A**) Carbon fixation pathways in prokaryotes were enriched in the NC group. (**B**) Selenocompound metabolism was enriched in the PG-diets group. The “+” sign outside the whisker boundaries is an outlier and the “star” sign represents the mean of the boxplot.

We performed a Phylogenetic Investigation of Communities by Reconstruction of Unobserved States (PICRUSt2) to predict functional pathway abundance related to PG-diets. Then, multiple groups comparison was computed using the STAMP software and the ANOVA statistical test. Out of 137 Kyoto Encyclopedia of Genes and Genomes (KEGG) pathways, 2 functional pathways were significantly abundant in the study (*p*-value < 0.05; Figure 5). The NC group showed enriched carbon fixation, whereas PG-diets showed enrichment in selenocompound metabolism.

## 3. Discussion

In this study, we demonstrated the effect of *P. grandiflorum* on the gut microbiome, especially in the immunosuppressed mouse model by CPA. The impact of the PG-diets on the altered gut microbiome in this study may support our previous study, which highlighted the preventive effect of PG and PGS extracts (PG-diets) on the immunosuppressed system in the NC group [16]. Furthermore, as a result of multiple analyses of gut microbiome and phenotype of immune-suppressed mice, our study provides a deeper insight into the relationship between the intake of *P. grandiflorum* root, microbial communities, and immune-related biomarkers.

The chemical which we use to suppress the immune system in mice is called CPA, and it is commonly used to treat repulsions and malignant tumors during organ or bone marrow transplants. However, CPA appeared to be noxious to normal cells, causing side effects such as weight loss, acute leukemia, liver dysfunction, anemia, hair loss, and severe problems arising from long-term use [23,24,25]. Thus, it is imperative to develop natural materials that can suppress the side effects of toxic immunosuppressants. In that sense, the roots of *P. grandiflorum* were considered suitable for inhibiting toxicity because it is known to contain large amounts of fibers, calcium, iron, and functional compounds such as saponins, which are commonly used to treat patients with bronchitis and asthma [26,27]. In addition, previous in vivo experiments reported that the root of PG improved the immunity of immunosuppressed mice at 150 mg/kg body weight [16]. However, the PG has a strong bitter taste which urged us to develop the aging red PG (PGS) that improved not only the taste but also the content of a physiologically active substance called platycodin D. Platycodin D has anti-cancer effects in addition to anti-inflammatory and diabetes prevention [28]. Moreover, it is well known that immunity can be improved by proliferating immune cells [29]. Thus, considering the PG with 150 mg/kg body weight showed an enhancing immunity in the in vivo experiment, PGS with 150 mg/kg and half of its amount (75 mg/kg) were evaluated to characterize the effect of PG. Our study revealed that supplements with PG-diets had an effect on reduced body weight loss in mice due to CPA treatment, where the body weight returned to that of the normal group. In addition, serum level of immunoglobulins (IgA and IgM) increased in the groups treated with each material without adverse or toxic effects after oral administration.

A decrease in body weight is considered as a side effect of long-term use of CPA treatment. The NC group showed a significant decrease in body weight compared to the Nor group, indicating that the side effects of CPA treatment appeared. According to the statistical *t*-test, the PG1 group presented a significantly increased body weight. Although PGS1 and PGS2 did not show a significant increase compared to the NC group, they displayed higher body weight than those of the NC and PC groups. In our in vivo study, PG-diets increased body weight that had decreased due to CPA, and a significant effect was found in the PG1 group compared to the NC group, which agrees with the observations in previous reports [16,20]. In addition, we observed that the concentration of IgA was significantly reduced in the NC group compared to the Nor group. However, their levels of PGS1, PGS2, and PG2 groups increased compared to the NC group (*p* > 0.05). A similar observation was shown in IgM, where the PG-diets groups leveled higher than the NC group. Therefore, our data suggest that CPA caused a significant reduction in body weight and immunoglobulin levels by its toxicity, while PG-diets can prevent the side effects of CPA and improve the conditions of mice treated with PG-diets. These findings indicate that the PG-diets can resist the immunosuppressive effects of CPA.

The gut microbiome diversity of seven groups elucidates the significant differences between groups. The microbiome in the Nor and NC groups was different, and the PG-diets groups, except for the PG2 group, were segregated with respect to the NC group, thereby indicating a distinctness between them. The altered abundance of bacteria following the PG-diets occurs at the generic level. The *Akkermansia* appeared to increase in the PG-diets. Similarly to the observation shown in body weight, the genus *Akkermansia* decreased in the NC group compared to the Nor group. Although the PC group supplemented with β-glucan shows a weak effect on the abundance, PG-diets elevated to a level similar to the Nor group. β-glucan is known to play a role in enhancing the immune system without becoming overactive, lowering elevated levels of low-density lipoprotein (LDL) cholesterol, and helping to prevent infections and cancer [30,31]. Interestingly, PG-diets may be more effective than β-glucan; however, this may result from the small sample sizes (*n* = 3~4) used in this experiment, which was a limitation. Moreover, previous papers reported that an increase in *Akkermansia* leads to anti-inflammatory activity in the intestinal tract and reduces diabetes, introduced as beneficial bacteria [32,33]. When bacteria strengthen the gut lining, improve metabolic health or support microbiome health, we call those bacteria beneficial bacteria [34]. Indeed, these have supported our results of presenting a positive correlation between body weight and *Akkermansia*. Thus, supplementing with PG-diets increased the abundance of *Akkermansia* and normalized the body weight with the Nor group compared to the NC group, indicating a favorable health effect. On the other hand, *Staphylococcus* is another significant microorganism at the general level, which showed a negative correlation between body weight. Its increasing trend was found in the NC group compared to the Nor group, whereas its decrease was found in the PGS group. However, no clear differences were observed between the PG-diets. In fact, *Staphylococcus* is known to cause food poisoning or skin allergy by its toxin. [2]. Therefore, the lower abundance of *Staphylococcus* is indicative of a healthier gut, supporting the idea of PG-diets altering the gut microbiome.

The association between microbiome and other traits, including IgA and IgM levels, was also determined, representing candidate microorganisms of its traits. Immunoglobulin levels were investigated as an index of the immune system related to the microbiome [35]. The immunoglobulin levels of PG-diets in the in vivo experiment were generally higher than those of the NC group (*p* > 0.05). Moreover, the genera associated with immunoglobulin levels were consistent with or contrary to previous metagenome studies related to the immune system. *Ruminococcaceae* and *Clostridium* groups that were negatively and positively correlated with IgA, respectively, were involved in inflammatory diseases. As *Ruminococcaceae* worked in the inoculation of strongly virulent inflammatory bowel disease, lower IgA level could be related to more abundant microorganism. In addition, functional analysis revealed that a combination of PG-diets were enriched in selenocompound metabolism. Selenium (Se) is an essential micronutrient for animals’ metabolism and is required for the biosynthesis of selenoproteins, which participate in the immune response, cancer chemoprevention, and other processes [36]. Therefore, the correlation between IgA level and enrichment of selenocompound metabolism suggests that altered communities of microorganisms induced by PG may alter the levels of immunoglobulin and be linked to the regulation of the immune system.

In our previous in vivo studies, PGS significantly improved immune-related biomarkers such as body weight, immunoglobulin, and NK cell activity than PG. Although we could not distinguish significant difference between PG and PGS on the gut microbiome and immune system, we have shown that PG-diets play a critical role in enhancing immunity and altering the gut microbiome. Therefore, future trials are recommended to evaluate their functional effects at various doses and supplemental periods with more sample sizes of mice and to develop suitable products for the people who need to improve their immune system by controlling the microbiome.

## 4. Materials and Methods

### 4.1. Preparations of Test Materials

The normal *Platycodon grandiflorum* (PG) and aged PG (PGS) extraction used in this experiment were provided by Hyundai Bioland (Ansan, Gyeonggi, Korea), which are strictly managed and produced according to production management standards. For PGS, it was prepared by washing the roots of domestic PG (Gumsan, Chungnam, Korea) twice, steaming them for 120 min, drying them for 24 h, repeating the steaming for 90 min 4 times, and drying them for 72 h to produce the red PGS. The weight of PGS or PG and 50% alcohol were added 15 times compared to the compounds and extracted for 8 h. The primary extract was recovered, and the remaining underwent secondary extraction with 50% alcohol for 8 h. All of the extracts were mixed and filtered using a filter press. It was concentrated under reduced pressure at a concentration of 60%, sterilized, and spray dried for the study. PG and PGS extracts were stored at 4 °C to protect from light and degradation until use.

### 4.2. Animals and Treatments

Six-week-old C57BL/6 male mice were supplied by Semtaco Co (Chunbuk, Korea). The mice were housed by 2 per polycarbonate cage in controlled conditions (20–25 °C, 50–55% humidity, and a 12 h light/dark cycle) with free access to water and standard rodent chow (38057, Purinafeed, Gyeonggi-do, Korea). After acclimation for 7 days, a total of 26 mice were randomly divided into 7 groups: (1) normal control (Nor); (2) cyclophosphamide (CPA) control (NC); (3) CPA + 2 mg β-glucan/body weight (PC) as a positive control of the preventive treatment; (4) CPA + 75 mg/kg body weight of PGS (PGS1); (5) CPA + 150 mg/kg body weight of PGS (PGS2); (6) CPA + 75 mg/kg body weight of PG (PG1); and (7) CPA + 150 mg/kg body weight of PG (PG2). The experimental extracts dissolved in distilled water were orally administered every day for two weeks. Mice of Nor and NC groups were administered with an equal volume of distilled water. Immunosuppression was induced by two intraperitoneal injections of CPA (Sigma-Aldrich, St. Louis, MO, USA). CPA was dissolved in saline, and 150 mg and 110 mg/kg of CPA were injected intraperitoneally 3 days and 1 day before treatment with PGS or PG, respectively, while the Nor group was injected with an equal volume of saline.

### 4.3. Metagenome Sequencing

Fecal samples were collected from each mouse and used for DNA extraction using AccuPrep Stool DNA Extraction Kit following the instructions of the manufacturer. After performing quality control (QC), qualified samples proceeded to library construction. The V3 and V4 region of the 16S rRNA genes were PCR amplified from the microbial genomic DNA. The DNA was quantified and qualified by agarose gel electrophoresis, Nanodrop Spectrophorometer (260/280 nm, 260/230 nm absorbance ratio) and Quant-iT™ PicoGreen™ dsDNA Assay Kit (Invitrogen). The input gDNA (10 ng) was PCR amplified using the barcoded fusion primers 341F/805R (341F: 5′ CCTACGGGNGGCWGCAG 3, 805R: 5′ GACTACHVGGGTATCTAATCC 3′). The final purified product was quantified using qPCR according to the qPCR Quantification Protocol Guide (KAPA Library Quantification kits for Illumina Sequencing platforms) and qualified using the LabChip GX HT DNA High Sensitivity Kit (PerkinElmer, Waltham, MA, USA). The 300 paired-end sequencing reaction was performed on the MiSeq™ platform (Illumina, San Diego, CA, USA). The sequencing data generated for this study are available at the Sequence Read Archive (SRA) of NCBI (http://www.ncbi.nlm.nih.gov/sra; Accessed on: 8 February 2021) under BioProject PRJNA700675.

### 4.4. Raw Data Processing and Taxonomic Analysis

Pre-processed reads were imported and analyzed using QIIME2 version 2020.02 [37]. We used the DADA2 software package implemented in QIIME2 to denoise and correct Illumina sequenced FASTA2Q files by removing chimera sequences using the “consensus” method [38]. Based on the quality plot of the DADA2, reads were trimmed using the following parameters: -*p*-trunc-len-f = 300; -*p*-trunc-len-r = 240; -*p*-trim-left-f = 6; and -*p*-trim-left-r = 6. For taxonomy assignment, a naïve Bayes classifier was trained on a GreenGenes 97% (version 13.8) operational taxonomic unit (OTU) database with reference sequences trimmed to the V3-V4 region using QIIME2 plugin feature-classifier [39].

### 4.5. Diversity Analysis

All of the sequence data were rarefied to a sampling depth of 12,361 sequences per sample prior to computation of alpha and beta-diversity analysis with QIIME2 plugin diversity, such as observed OTUs, Shannon, and weighted Unifrac. The weighted Unifrac distance matrix was used for nonparametric Permutation Multivariate Analysis of Variance (PERMANOVA) and Principal Coordinate Analysis (PCoA) plot [40,41,42,43]. PER-MANOVA was performed with 999 permutations to weighted UniFrac distance matrix using Adonis function in R package ‘vegan’ [44].

### 4.6. Performance Analysis

In the present study, body weight and serum immunoglobulin levels of experimental mice were measured. A total of 26 samples (3 or 4 mice in each of the 7 groups) similar to the average group weight were selected for the performance analysis. Body weights were monitored once a week. All animals were overnight fasted (water was not restricted) before initial test substance administration and sacrifice to re-duce the individual differences from feeding. The mice were sacrificed under inhalation anesthetized with CO_2_, using rodent inhalation anesthesia apparatus (Surgivet, Waukesha, WI, USA). Serum concentrations of immunoglobulin A (IgA) and immunoglobulin M (IgM) were measured using enzyme-linked immunosorbent assays kits (ELISA; Abcam, Cambridge, UK), according to the manufacturer’s instructions. All of the assays were performed in duplicate. All of the animals were treated according to the international regulations for the usage and welfare of laboratory animals. This study was approved by the Institutional Animal Care and Use Committee in the National Institute of Agricultural Sciences (NAS-201807).

### 4.7. Statistical Analysis

Trimmed Mean of M values (TMM) was obtained to adjust for different library sizes using edgeR [45]. Then, statistical tests were performed under a generalized linear model (GLM), considering OTU’s count as a negative binomial distribution. To compare the goodness-of-fit of 2 models, the log-likelihood ratio statistic was calculated, and the false discovery rate (FDR) was used to adjust for multiple testing errors with a significance level of 5% [46]. Another statistical test for differentially abundant microbial taxa was assessed using the QIIME2 Analysis of Composition of Microbiomes (ANCOM) plugin in order to identify features that significantly differed in abundance from each study group [47]. The differentially abundant features at each phylogenetic level were calculated by ANCOM from the DADA2 feature table. The Student’s *t*-test was used to compare the biomarkers (body weight, IgA, and IgM) between groups. Moreover, the pairwise correlations between the abundance of microbiome and immune-related biomarkers were determined using Spearman correlation coefficient (r) from the *corplot* R package. The abundance of significantly correlated genera (*p*-value < 0.05) was visualized in a line graph with the corresponding phenotype to see its correlations. All of the R packages were implemented in RStudio version 4.0.1 [48].

### 4.8. Functional Profiles of Gut Microbiome

Phylogenetic Investigation of Communities by Reconstruction of Unobserved States2 (PICRUSt2) was used to predict Kyoto Encyclopedia of Genes and Genomes (KEGG) Orthology (KO) genes from 16S rRNA data [49]. The tree generated in PICRUSt2 with the maximum nearest-sequenced taxon index (NSTI) cutoff of 2 was complemented with the feature table resulted from QIIME2 plugin dada2 for hidden-state prediction (hsp) using the maximum-parsimony method [50]. Followed by the prediction of KO genes, the KEGG pathways were then mapped to the KO genes using the PICRUSt2 package. Afterward, multiple group comparison was computed using the Analysis of Variance (ANOVA) statistical test (*p*-value < 0.05) in the STAMP software package to carry out significant pathways [51]. Moreover, functions related to human diseases were achieved using White’s nonparametric *t*-test from the STAMP software package [52].

## 5. Conclusions

Our results demonstrate the extent to which PG supplementation in a mouse model of immunosuppression alters the gut microbiome and host phenotypes, namely body weight and IgA levels. Although the study was limited by the small sample size and amount of intake, this was the first study to investigate the effect of PG on the gut microbiome and immune system, despite several studies reporting its health benefits. The abundance of Akkermansia increased in the immunodeficient mouse supplemented with PG-diets, indicating that the PG has a distinct effect on microbial communities. Furthermore, we investigated specific genera related to body weight and serum immunoglobulin levels, which were known to be important for the immune system. As PG-diets have provided a functional compound related to enhancing immunity, the health benefit of PG-diets in immunodeficiency may mediate via its microbiome, such as *Akkermansia* microorganism. Moreover, PG-diets could be a potential nourishing immunity supplement for an immunosuppressed individual. In addition, PG-diets could contribute to improving the value of domestic PG, as well as enhancing the reliability of domestic foods.

## Data Availability

The sequencing data analyzed for this study are available via the NCBI Sequence Read Archive (SRA) under accession number BioProject PRJNA700675.

## Supplementary Materials

The following are available online at https://www.mdpi.com/article/10.3390/metabo11120817/s1. Figure S1: Observed OTUs and Shannon index in seven groups, Figure S2: Differentially abundant microbial taxa identified by q2-ANCOM, Figure S3: Correlation between IgA and IgM levels and relative abundance of microorganisms.
