# Supplementary material for: Effects of Platycodon grandiflorum on Gut Microbiome and Immune System of Immunosuppressed Mouse
Source: Metabolites. 2021 Nov 29;11(12):817. doi: 10.3390/metabo11120817 (PMC8707369; doi:10.3390/metabo11120817)
Supplement: Supplementary file 1 [file metabolites-11-00817-s001.zip › metabolites-1485124-supplementary.pdf]

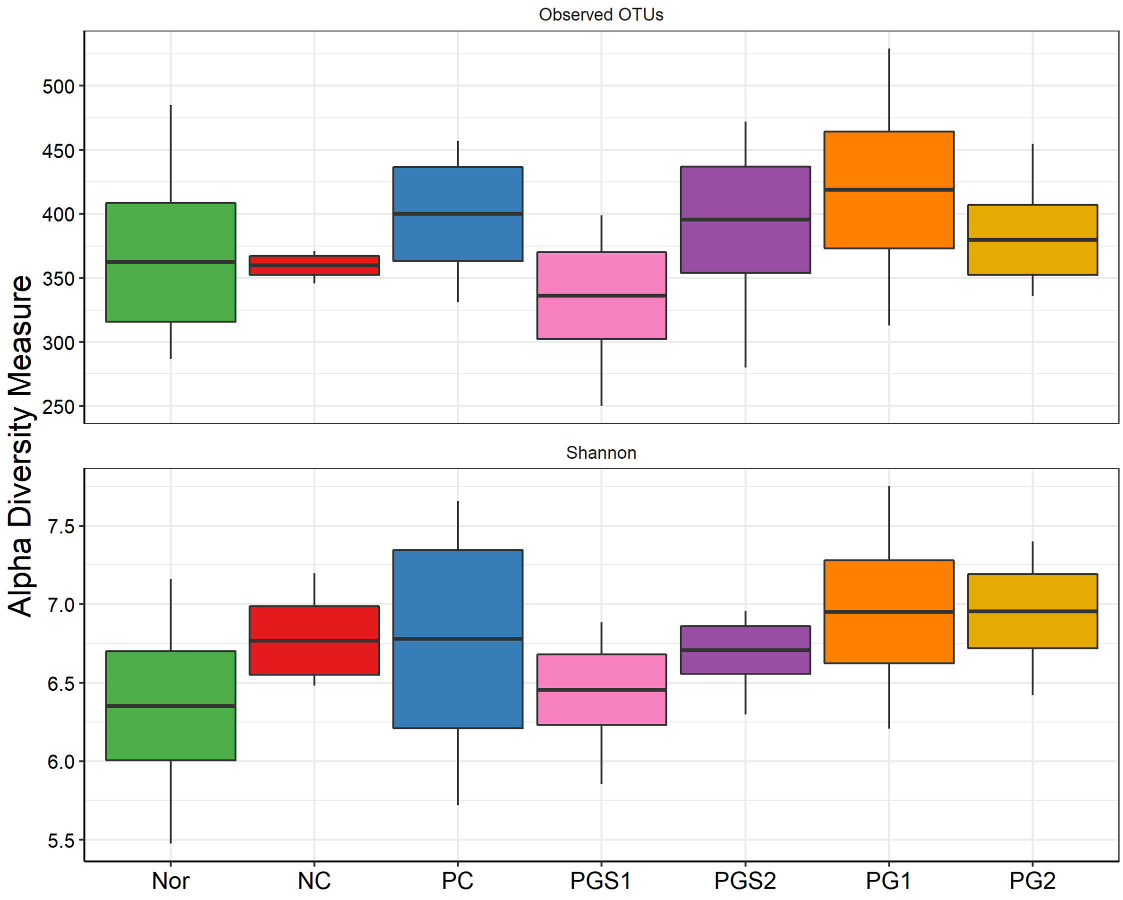

Figure S1. Observed OTUs and Shannon index in seven groups. The x-axis represents seven groups and the y-axis presents the alpha diversity measurements: observed OTUs and Shannon index.

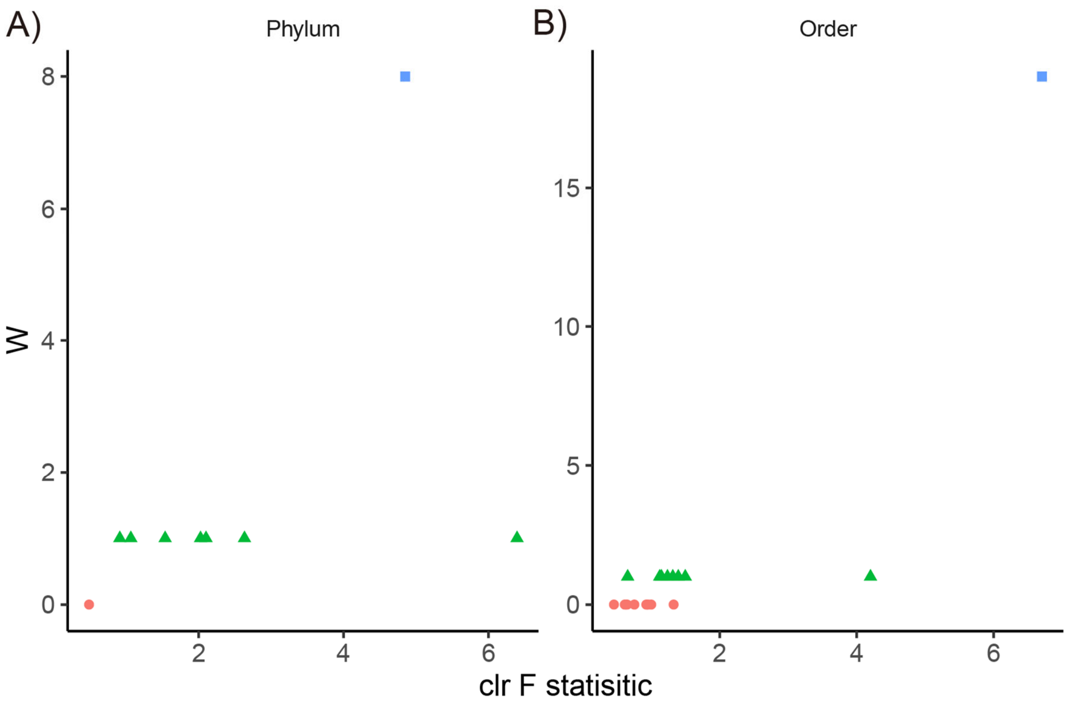

Figure S2. Differentially abundant microbial taxa identified by q2-ANCOM. ANCOM volcano plot of differential abundance at the group level, where the x-axis represents F-statistics and y-axis represents the W-statistics. A) At the phylum level, the square represents *Verrucomicrobia*, the triangle represents *Bacteria*, *Actinobacteria*, *Bacteroidetes*, *Firmicutes*, *Fusobacteria*, *Proteobacteria*, and *Tenericutes*, circle represents *Deferribacteres*. B) At the order level, the square represents *Verrucomicrobiales*, the triangle represents *Bacteroidales*, *Bacillales*, *Gemellales*, *Lactobacillales*, *Clostridiales*, *Fusobacteriales*, *Pseudomonadales*, and *RF39*, circle represents *Actinomycetales*, *Coriobacteriales*, *Deferribacterales*, *Turicibacterales*, *Erysipelotrichales*, *Burkholderiales*, *Desulfovibrionales*, and *Anaeroplasmatales*.

A)

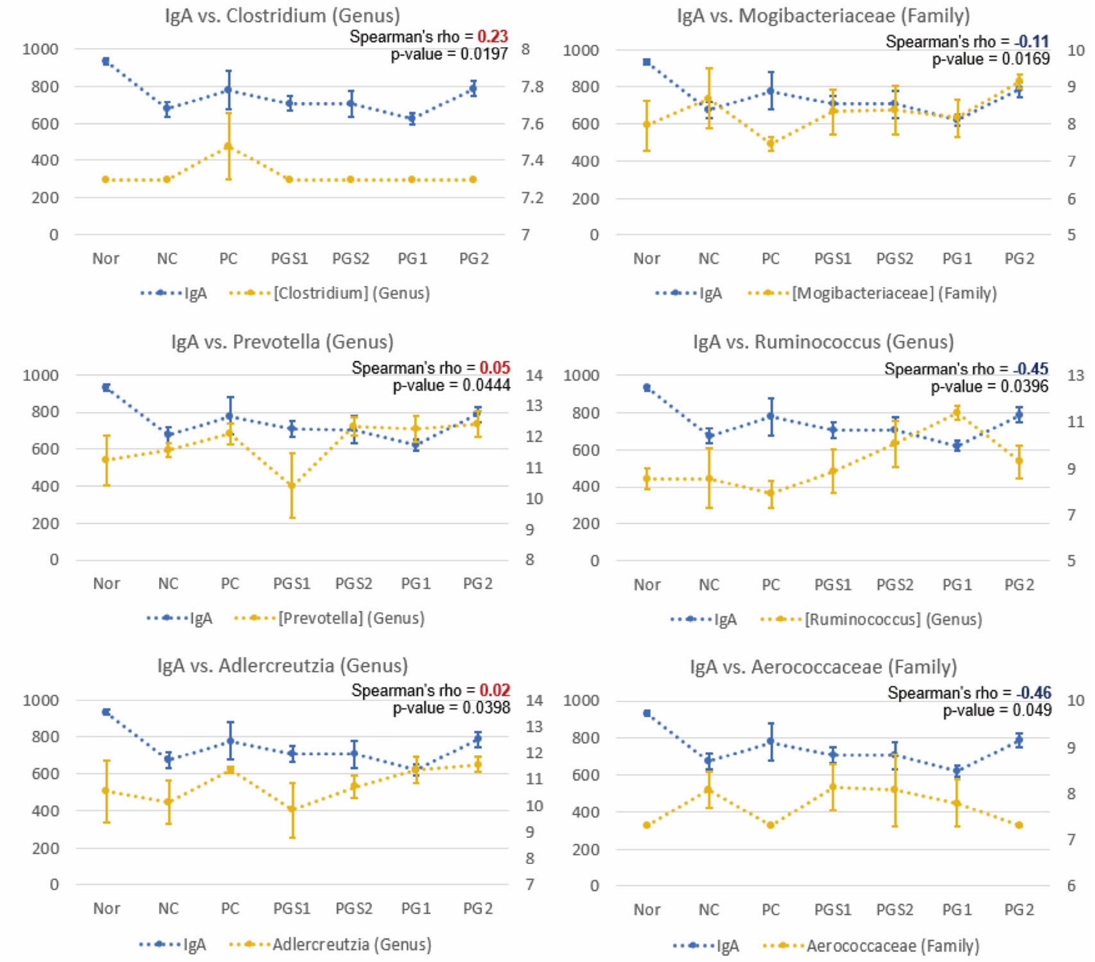
IgA vs. Clostridium (Genus)
Spearman's rho = 0.23
p-value = 0.0197
IgA
[Clostridium] (Genus)
IgA vs. Mogibacteriaceae (Family)
Spearman's rho = -0.11
p-value = 0.0169
IgA
[Mogibacteriaceae] (Family)
IgA vs. Prevotella (Genus)
Spearman's rho = 0.05
p-value = 0.0444
IgA
[Prevotella] (Genus)
IgA vs. Ruminococcus (Genus)
Spearman's rho = -0.45
p-value = 0.0396
IgA
[Ruminococcus] (Genus)
IgA vs. Adlercreutzia (Genus)
Spearman's rho = 0.02
p-value = 0.0398
IgA
Adlercreutzia (Genus)
IgA vs. Aerococcaceae (Family)
Spearman's rho = -0.46
p-value = 0.049
IgA
Aerococcaceae (Family)
Nor
NC
PC
PGS1
PGS2
PG1
PG2

B)

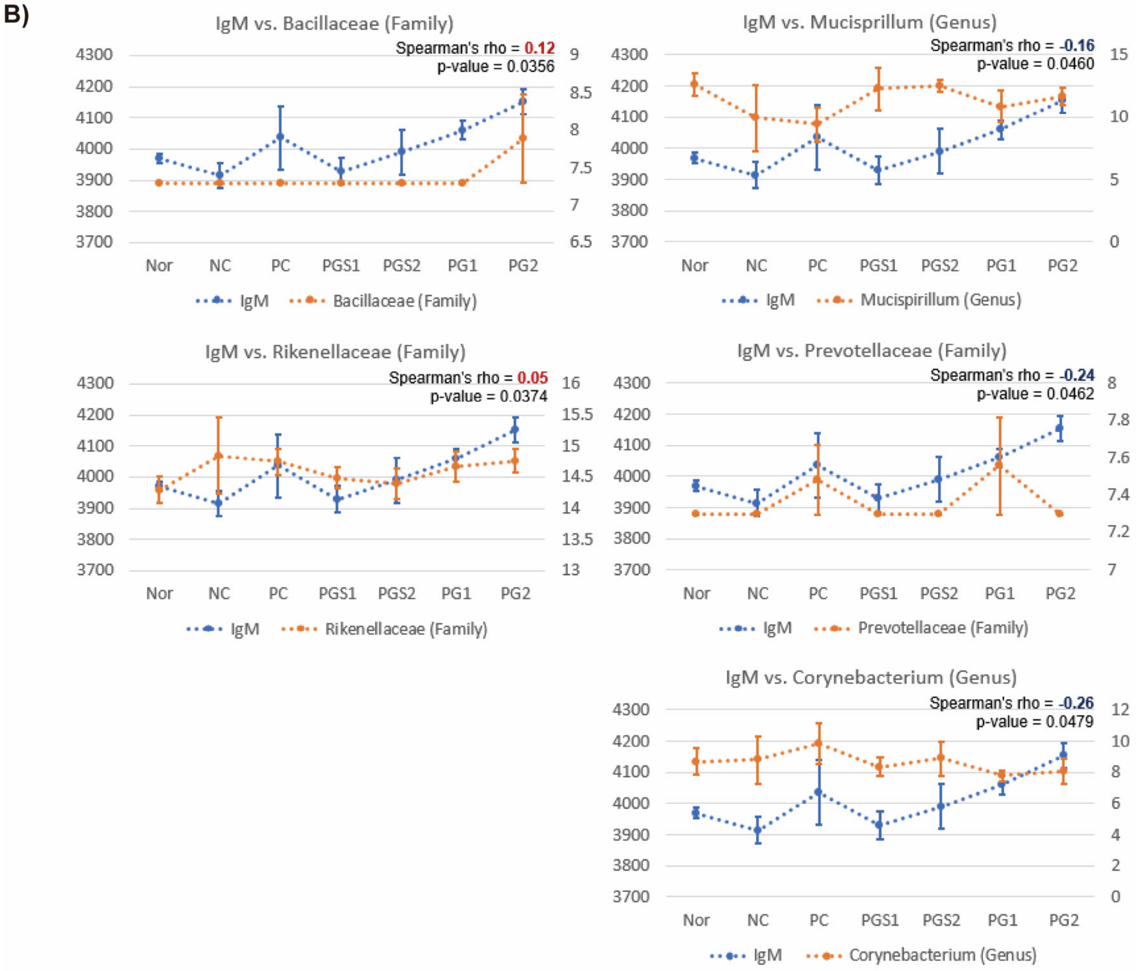

Figure S3. Correlation between IgA and IgM level and relative abundance of microorganisms. Total of six and five significant genera were correlated with IgA and IgM, respectively (A, B). The x-axis is the group, and the left y-axis is the phenotype values (blue) and right y-axis is the relative abundance values (IgA: yellow; IgM: orange). The Spearman’s coefficient and p-value are placed on the right top corner.
